# Fungal Pathogen Infection by *Metarhizium anisopliae* Alters Climbing Behavior of *Lymantria dispar* with Tree-Top Disease Induced by LdMNPV

**DOI:** 10.3390/biology14081029

**Published:** 2025-08-11

**Authors:** Qi Song, Yu-Shan Wei, Dun Wang

**Affiliations:** 1 Key Laboratory of Plant Protection Resources and Pest Management of Ministry of Education, College of Plant Protection, Northwest A&F University, Yangling 712100, China; songsongee@foxmail.com (Q.S.); weiyushan123@foxmail.com (Y.-S.W.); 2 Key Laboratory of Integrated Pest Management on the Loess Plateau of Ministry of Agriculture and Rural Affairs, Northwest A&F University, Yangling 712100, China

**Keywords:** tree-top disease, behavior change, *Metarhizium anisopliae*, *Lymantria dispar*, LdMNPV

## Abstract

Certain parasites manipulate host behavior following infection to enhance their own dispersal and transmission. Lepidopteran larvae infected with baculoviruses exhibit increased locomotion, ascending to the apex of their host plant where they ultimately die in a characteristic inverted, liquefied posture suspended by their prolegs—a phenomenon termed “tree-top disease”. Although numerous studies have investigated the underlying causes of this behavior, the precise mechanism governing tree-top disease formation remains unresolved. In this study, *Lymantria dispar* larvae were infected with *Metarhizium anisopliae* and Lymantria dispar multiple nucleopolyhedrovirus (LdMNPV). We compared symptom profiles across infection modes and assessed virulence, demonstrating that *M. anisopliae* infection alters the hyperactive state induced by LdMNPV in larvae exhibiting tree-top disease. Specifically, *M. anisopliae* promoted tree-top disease behavior during early infection stages but suppressed it during later stages. Furthermore, the symptomatology of larvae co-infected with both pathogens differed significantly from that observed in larvae infected with either *M. anisopliae* or LdMNPV alone. Co-infected larvae also exhibited accelerated mortality compared to those infected with a single pathogen. The above findings indicate that *L. dispar* larvae, when co-infected with LdMNPV and *M. anisopliae* may change behavioral responses that could further modulate the pathogenesis of LdMNPV-induced tree-top disease. Furthermore, a synergistic interaction between *M. anisopliae* and LdMNPV was observed in the biocontrol of *L. dispar*.

## Simple Summary

This study investigated the “tree-top disease” behavior of gypsy moth larvae infected by *Metarhizium anisopliae* and Lymantria dispar nucleopolyhedrovirus (LdMNPV) under different infection regimes. The results revealed that *M. anisopliae* infection altered the hyperactive state of larvae with tree-top disease that induced by LdMNPV. Notably, the symptoms in gypsy moth larvae co-infected with both *M. anisopliae* and the virus differed significantly from those infected with either pathogen alone. Moreover, larvae with dual infection exhibited earlier mortality compared to those infected solely with *M. anisopliae* or the virus. These findings indicate that *M. anisopliae* can influence the virus-induced tree-top disease in gypsy moth, and a synergistic effect exists between *M. anisopliae* and the virus in controlling gypsy moth. The above conclusions deepen our understanding of the underlying mechanisms of tree-top disease in gypsy moth and suggest a novel approach for its biocontrol via the combined application of entomopathogenic fungi and viruses.

## 1. Introduction

*Lymantria dispar*; commonly known as the Asian gypsy moth, is a globally distributed and severe forest pest belonging to the family Erebidae within the order Lepidoptera. Its larvae are broadly polyphagous, feeding on a wide range of host trees including oaks, deciduous hardwoods, and some conifers, with a preference for new leaves or buds [1,2,3,4]. Baculoviruses are a category of obligate arthropod pathogens that exhibit a global distribution, primarily infecting insects within the orders Lepidoptera, Diptera, and Hymenoptera [5,6,7]. Their genomes consist of large, double-stranded, circular DNA molecules and exhibit significant genetic diversity among different species [6,8]. According to the most recent taxonomic classification, baculoviruses are categorized into genera such as Alphabaculovirus, Betabaculovirus, and Gammabaculovirus. For instance, a virus isolated from *Nesodiprion zhejiangensis* has been classified as a novel Gammabaculovirus [9,10]. Specific groups of baculoviruses can manipulate insect hormone metabolism via ecdysteroid UDP-glucosyltransferase (EGT), inducing pronounced aberrant behavioral phenotypes in infected hosts [11,12]. Lepidopteran insects infected by such baculoviruses exhibit vertical migratory behavior, ascending from their normal habitats towards the tree canopy or apex. They subsequently die in a characteristic inverted posture anchored by their prolegs beneath leaves, undergoing tissue liquefaction and cuticular disintegration. Upon death, they release viral particles. This pathological manifestation is collectively termed “tree-top disease” in the field of insect virology [12,13]. The application of both Lymantria dispar multiple nucleopolyhedrovirus (LdMNPV) and Lymantria dispar multiple cypovirus (LdMCPV) to infect *L. dispar* larvae in experimental settings has been demonstrated to show potential for control efficacy. Notably, LdMNPV-infected *L. dispar* exhibits the aforementioned “tree-top disease” characteristics [14,15].

The pathogenic mechanisms underlying tree-top disease in Lepidoptera remain incompletely elucidated. Recent research indicates that its development primarily involves several key elements, with hormonal signaling playing a central regulatory role alongside the involvement of specific genes and signaling pathways, while light exposure serves as a critical triggering factor. Juvenile hormone (JH) and 20-hydroxyecdysone (20E) have been identified as key signaling molecules inducing the disease. RNAi-mediated knockdown of JH or exogenous JH application demonstrates that JH mediates the upward migratory behavior of larvae, whereas 20E suppresses disease occurrence by antagonizing JH effects. BrZ2, acting as a downstream target of JH/20E signaling and regulated by two miRNAs, enhances Helicoverpa armigera nucleopolyhedrovirus-induced tree-top disease when knocked down [16]. Following LdMNPV infection in *L. dispar* larvae, significant alterations in host gene expression occur from the asymptomatic phase to the tree-top disease stage. Pathway enrichment analysis identified 34 signaling mechanisms, suggesting a potential critical role for the PI3K/AKT signaling pathway and associated genes in disease pathogenesis [17,18]. Crucially, tree-top disease is induced only by overhead light exposure (e.g., blue light 450–490 nm, UVA 320–400 nm, and white light), with illumination from below proving ineffective [19,20]. In *Spodoptera exigua* larvae infected with Spodoptera exigua nucleopolyhedrovirus, light exposure must occur within a specific 43–50 h post-infection window to trigger the behavioral symptoms; larvae exposed earlier than this critical period or deprived of light ultimately succumb at lower positions [20].

The interaction effects of baculoviruses with other pathogens primarily manifest in co-infections involving other viruses, bacteria, or fungi. When baculoviruses co-infect insect hosts alongside other baculoviruses, this interaction can increase viral lethality, thereby accelerating host death [7]. The co-infection of insect hosts with baculoviruses and *Bacillus thuringiensis* (Bt) has been shown to significantly enhance insecticidal efficiency. This phenomenon may be attributed to the disruption of midgut structure, as observed in previous studies [21,22]. In cases of co-infection with certain fungal pathogens, baculoviruses and fungi exhibit convergent evolution, utilizing distinct molecular mechanisms to cooperatively manipulate host behavior. This manipulation induces the manifestation of “tree-top disease,” driving the host to climb to elevated positions, which facilitates more effective pathogen dispersal [11]. The regulatory mechanisms by which fungi modulate insect behavior are complex and multifaceted, primarily rooted in their interactions with host organisms, particularly entomopathogenic fungi and plant-symbiotic fungi [23,24,25]. These mechanisms primarily operate through the alteration of chemical signaling, manipulation of physiological responses, or exertion of plant-mediated indirect effects, aimed at facilitating fungal dissemination, evading host immunity, or optimizing the infection process [26,27,28]. In the present study, we employed laboratory virulence assays to elucidate the biocontrol efficacy of individual applications and co-infection with both the virus and the fungus. Furthermore, an investigation was conducted into the disparities in climbing behavior induced by co-infection in comparison to single infections. This study aimed to explain the mechanisms of tree-top disease while offering critical insights into its relevance to biocontrol.

## 2. Materials and Methods

### 2.1. Insects, Virus, and Spore Suspension

*L. dispar* larvae were continuously maintained on an artificial diet in climate-controlled chambers (Model YKGC-500L, Youke Instrument & Equipemnt Co., Ltd., Hefei, China) at 25 ± 1 °C, 60 ± 5% RH, 14L:10D photoperiod through successive generations.

LdMNPV stocks were maintained in the laboratory. Occlusion bodies (OBs) were extracted from liquefied larvae using the following protocol: Infected larvae were homogenized in 1× phosphate-buffered saline (PBS), filtered through multilayer gauze, and subjected to repeated centrifugation at 6000 rpm by a centrifuge (Model 5424R, Eppendorf, Hamburg, Germany) with PBS washes until the supernatant clarified. The resulting pellet was resuspended in PBS to constitute the LdMNPV stock solution. The resuspended precipitate was quantified using a bacterial counting chamber and then diluted to a final concentration of 10^9^ OBs/mL. The suspension was stored at 4 °C for further use.

The entomopathogenic fungal strain *Metarhizium anisopliae* HJN-G3-2C—originally isolated from soil via *Tenebrio molitor* baiting and selected for enhanced virulence through preliminary pathogenicity screening—was cryopreserved at −80 °C in Panasonic Freezer (Model MDF-382, Sonyo Refrigeration (Dalian), Co., Ltd., Dalian, China). For activation, stock cultures were incubated on potato dextrose agar (PDA) under controlled conditions (25 ± 2 °C, 70 ± 5% RH, 12L:12D photoperiod) in an Incubator (Model SPX-420B, Shanghai Nanrong Laboratory Equipment Co., Ltd., Shanghai, China). Conidia were collected at 14 days post-inoculation. Spore viability was assessed using a dual-staining system comprising 0.05% (*v*/*v*) fluorescein diacetate (FDA) and 50 μg/mL propidium iodide (PI). A conidial suspension was prepared by homogenization in 0.05% (*v*/*v*) Tween-80 solution.

Both pathogen preparation protocols followed established methodologies previously validated in peer-reviewed studies [29,30].

### 2.2. Virulence Assay of LdMNPV Infection in L. dispar

LdMNPV suspensions at concentrations of 2 × 10^5^, 2 × 10^6^, 2 × 10^7^, 1 × 10^8^, and 2 × 10^8^ OBs/µL were prepared as described previously. A 20% (*w*/*v*) sucrose solution was also prepared. The different concentrations of LdMNPV suspension were mixed with the 20% sucrose solution at a 1:1 (*v*/*v*) ratio. Following a starvation pretreatment, the 3rd-instar larvae of *L. dispar* were individually administered 2 µL aliquots of varying concentrations of LdMNPV solution using the droplet-feeding method [31]. Mortality was recorded at 24 h intervals, and the status of larval death was noted, until all larvae had died. Each treatment consisted of 24 larvae, and the experiment was replicated three times.

### 2.3. Virulence Assay of M. anisopliae Infection in L. dispar

*M. anisopliae* strain HJN-G3-2C conidial suspensions at concentrations of 5 × 10^5^, 5 × 10^6^, 5 × 10^7^, 5 × 10^8^, and 5 × 10^9^ conidia/mL were prepared as described previously. Suspensions were loaded into individual spray bottles. The 3rd-instar larvae of *L. dispar* were topically treated by sequential spraying with each concentration, ensuring complete conidial coverage on the larval cuticle [32]. Control groups received 0.05% (*v*/*v*) Tween-80 in sterile water instead of conidial suspension. Mortality was recorded at 24 h intervals with documentation of lethal manifestations until 100% mortality was achieved. Cadavers were transferred to the chambers (Model YKGC-500L, same as above), maintained with sterile water-moistened filter paper within the rearing containers, for incubation. Each treatment utilized 24 larvae with three experimental replicates.

### 2.4. Virulence Assay of L. dispar Larvae Under Co-Infection

The 3rd-instar larvae of *L. dispar* were first treated with the viral solution according to the method described above. Following the completion of feeding on the viral solution, a fungal spore suspension was then sprayed onto the larvae using the aforementioned method. The control group consisted of larvae subjected to starvation, subsequently fed 2 μL of a 20% sucrose solution, and then sprayed with a sterile aqueous solution containing 0.05% (*v*/*v*) Tween-80. Mortality was recorded at 24 h intervals, along with observations of larval death status. Monitoring continued until all larvae had died. Cadavers were placed in humidified chambers for incubation, with moisture maintained using sterile water-moistened filter paper within the rearing containers. The treatment group comprised 24 larvae, and the entire experiment was performed in three independent replicates.

### 2.5. Vertical Displacement Differences in L. dispar Larvae Following Co-Infection

The 3rd-instar larvae of *L. dispar* were treated according to the method described above. Climbing height over a 15 min period and mortality were recorded at 24 h intervals (5000 Lux, 14L:10D photoperiod, 25 ± 1 °C). Climbing height was measured using a specialized vertical displacement chamber designed previously [22]. Measurements were conducted at a fixed time daily, and the entire experiment was performed in three replicates.

### 2.6. Statistical Analyses

For the virulence bioassay, mortality and corrected mortality rates for each treatment were calculated using SPSS 25.0 software. A toxicity regression equation, along with the median lethal concentration (LC_50_) and lethal concentration 90 (LC_90_) values, were determined via probit analysis, where the independent variable (X) was the logarithm of the spore suspension concentration (spore/mL) and the dependent variable (Y) was the probit value of the mortality rate.

For the climbing behavior assay, experimental data were analyzed and relevant graphs were generated using GraphPad Prism 8. Statistical analysis was performed using one-way ANOVA in SPSS 25.0, followed by Duncan’s multiple range test for post hoc comparisons. Differences were considered statistically significant at *p* < 0.05.

All the aforementioned statistical methods involve normality tests and homogeneity of variance tests to estimate parameters and compare intergroup differences, thereby ensuring the accuracy of statistical inferences.

## 3. Results

### 3.1. Death Symptoms of L. dispar Larvae Infected with LdMNPV and M. anisopliae

**Figure 1:**
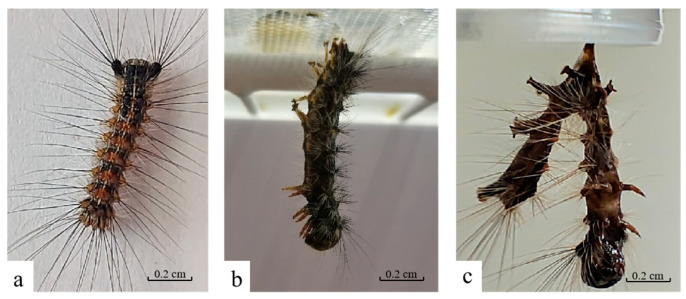
The death symptoms of *L. dispar* larvae infected by LdMNPV. (**a**) 4 d; (**b**) 6 d; (**c**) 8 d.

During post-infection days 1 to 3, no significant differences were observed between LdMNPV-infected larvae and healthy larvae. By post-infection day 4, the virus-infected larvae exhibited increased activity but displayed slowed feeding and growth (Figure 1a). On post-infection day 6, the infected larvae climbed to the top of the rearing container and ultimately died while suspended by their prolegs, undergoing liquefaction (Figure 1b). Three days post-mortem, the cadavers ruptured, releasing LdMNPV into the external environment (Figure 1c).

**Figure 2:**
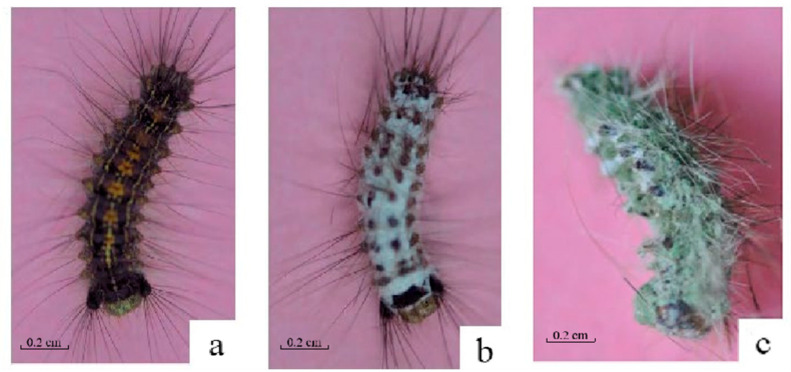
The death symptoms of *L. dispar* larvae infected by Strain HJN-G3-2C of *M. anisopliae*. (**a**) 4 d; (**b**) 6 d; (**c**) 8 d.

For *M. anisopliae* strain HJN-G-3-2C infection, from 1 d to 3 d, larvae showed no observable differences in external morphology or feeding behavior relative to healthy controls, except for reduced food consumption (Figure 2a). Larval mortality typically occurred around 4 d post-infection. By 3 d post-mortem, dense white mycelia had colonized the cadaver surface (Figure 2b). Partial sporulation was evident by 5 d post-mortem, and distinct sporulation became apparent, with green conidia emerging from the mycelial mat. The coloration of the conidial layer progressively deepened from light green to dark green (Figure 2c).

### 3.2. Death Symptoms of L. dispar Larvae Under LdMNPV and M. anisopliae Co-Infection

**Figure 3:**
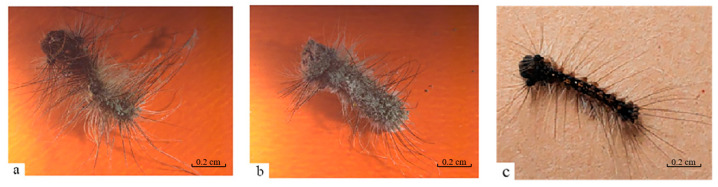
The death symptoms of *L. dispar* larvae infected by *M. anisopliae* and LdMNPV. (**a**) Co-infected by strain HJN-G3-2C of *M. anisopliae* and LdMNPV; (**b**) infected by strain HJN-G3-2C of *M. anisopliae*; (**c**) infected by LdMNPV.

Cadavers of *L. dispar* larvae in the treatment group co-infected with the *M. anisopliae* strain HJN-G3-2C and LdMNPV were desiccated, developed white mycelia on their surface, and subsequently exhibited a change in spore color from light green to dark green (Figure 3a). In contrast, cadavers of larvae in the control group infected solely with the *M. anisopliae* strain HJN-G3-2C remained relatively plump, although they also developed white mycelia and green spores (Figure 3b). Similarly, cadavers of larvae in the control group infected solely with LdMNPV darkened post-mortem and showed no development of mycelia or spores (Figure 3c).

### 3.3. Virulence of LdMNPV and M. anisopliae Against L. dispar Larvae

At 10 d post-application, *M. anisopliae* strain HJN-G-3-2C exhibited an LC_50_ of 1.77 × 10^8^ spore/mL and an LC_90_ of 2.501 × 10^10^ spore/mL (Table 1). Both LT_50_ and LT_90_ values demonstrated a concentration-dependent decline. At a spore concentration of 5 × 10^7^ spore/mL, the LT_50_ and LT_90_ values for *M. anisopliae* HJN-G-3-2C against *L. dispar* larvae were 8.373 d and 16.705 d, respectively. Conversely, under viral challenge at 2 × 10^7^ OBs/mL, LdMNPV yielded LT_50_ and LT_90_ values of 5.457 d and 11.920 d, respectively (Table 2). The data presented above demonstrate that the LdMNPV formulation, at concentrations with significantly lower numerical values than those of *M. anisopliae* strain HJN-G3-2C conidia, induced significantly shorter LT_50_ and LT_90_ values against *L. dispar* larvae.

### 3.4. Virulence of LdMNPV and M. anisopliae Co-Infection Against L. dispar Larvae

**Figure 4:**
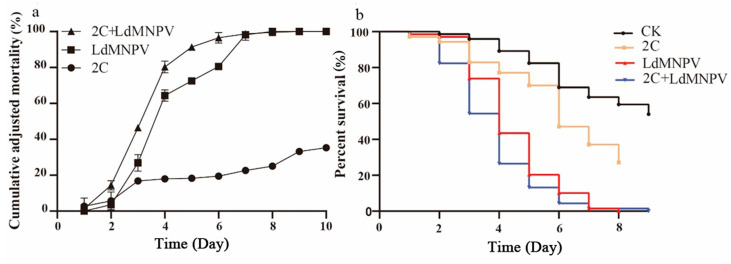
The cumulative adjusted mortality and percent survival of 2C and LdMNPV. (**a**) Cumulative adjusted mortality; (**b**) percent survival. Note: 2C: Strain HJN-G3-2C of *M. anisopliae*.

Co-infection assays were conducted using *M. anisopliae* strain HJN-G-3-2C at 1 × 10^8^ spore/mL and LdMNPV at 1 × 10^8^ OBs/mL. Larval mortality increased progressively over time, with discernible differences between single and co-infection treatments emerging by 3 d post-infection. At 4 d post-infection, co-infected *L. dispar* larvae exhibited approximately 80% mortality, matching the mortality rate in LdMNPV-alone treatment, whereas *M. anisopliae* mono-infection yielded only 20% mortality. By 8 d post-infection, both viral single infection and co-infection groups reached 100% mortality (Figure 4a). Statistical analyses revealed significant differences between LdMNPV-infected and control groups at 3 d post-infection, with co-infection also differing significantly from controls. Peak mortality in the co-infection group occurred at 6 d post-infection, demonstrating statistically distinct lethality compared to all other treatments. All experimental groups differed significantly from controls at this timepoint, collectively demonstrating accelerated mortality under co-infection conditions (Figure 4b).

### 3.5. Climbing Speed of L. dispar Larvae Under LdMNPV and M. anisopliae Co-Infection

**Figure 5:**
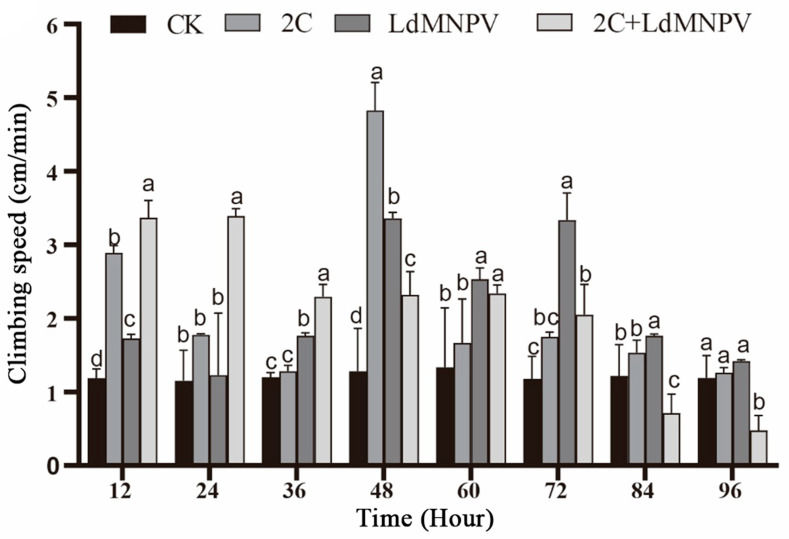
Climbing speed after mixed infection with 2C and viruses. Note: 2C: Strain HJN-G3-2C of *M. anisopliae*. Lowercase letters designate a significant difference among different tissues at the same timepoint. (*p* < 0.05). Data are shown as means ± standard errors (SEs).

Significant temporal variations in larval climbing speed were observed across infection treatments. At 12 h post-infection, co-infected larvae exhibited peak climbing velocity, followed by those infected with *M. anisopliae* strain HJN-G-3-2C. Co-infected larvae maintained superior climbing speed relative to other groups at 24 h post-infection, though all treatments showed progressive decline beyond this timepoint. Maximum climbing speed (4.8 cm/min) was recorded in *M. anisopliae*-infected larvae at 48 h post-infection. By 72 h post-infection, the LdMNPV mono-infection group demonstrated the highest climbing velocity, whereas co-infected larvae displayed markedly reduced locomotion. Notably, climbing speed in *M. anisopliae*-infected and control groups showed no significant divergence at this terminal observation (Figure 5). The overall attenuation of climbing performance at 72 h post-infection is likely attributable to larval molting progression. Despite maintaining comparative advantage over other treatments, LdMNPV-infected larvae exhibited slower climbing speeds relative to the 48 h timepoint.

## 4. Discussion

This study demonstrates that *M. anisopliae* co-infection exerts a biphasic effect on tree-top disease behavior in *L. dispar* larvae. At 24 h post-infection, climbing speed in fungus-infected larvae significantly exceeded that of both controls and LdMNPV mono-infected groups. By 72 h post-infection, however, co-infected larvae exhibited markedly reduced climbing speed compared to virus-only infected counterparts. This indicates that fungal infection initially promotes tree-top disease behavior during early infection but suppresses it in later stages. This phenomenon of initial stimulation followed by inhibition of host locomotion parallels observations of *M. anisopliae* modulating movement in *Reticulitermes chinensis* and *Solenopsis invicta* [33,34]. The biphasic “low-promotion, high-inhibition” effect may be associated with insect immune or behavioral compensatory mechanisms. Following fungal challenge, insects concurrently alter immune and behavioral responses, integrating these into a distinct bio-behavioral compensatory system [26,35]. Entomopathogenic fungi precisely regulate host behavioral genes to manipulate this system, thereby enhancing host mobility and wandering behavior [36,37]. This culminates in induced climbing and attachment to elevated vegetation, followed by progressive loss of motor function until death—a strategy ensuring optimal spore dispersal [38,39,40]. These findings provide empirical support for the proposed hypothesis while suggesting novel research avenues for elucidating tree-top disease mechanisms. Future investigations should target genes integral to the insect bio-behavioral compensatory system, such as tyrosine hydroxylase (*TH*) and dopamine *N*-acetyltransferase (*Dat*) [41,42].

Current evidence indicates that genes within the insect bio-behavioral compensatory system govern locomotion primarily through dopaminergic pathways. For instance, in *Bombyx mori*, viral infection triggers specific overexpression of TH in the brain, resulting in significantly elevated dopamine levels. This increase subsequently enhances locomotor and foraging activities. Crucially, administering a TH inhibitor blocks this virus-induced hyperlocomotion, confirming TH’s direct role in modulating motor activity via dopamine-dependent mechanisms [43]. Dat catalyzes the Ac-CoA-dependent acetylation of dopamine to form *N*-acetyldopamine. This reaction inactivates the neurotransmitter, directly modulating the intensity of dopaminergic signaling and thereby influencing diverse behaviors including locomotion, learning, memory, and stress responses [44,45,46]. However, the fundamental mechanisms for TH/Dat regulation of dopamine pathways are still unclear. Further studies were needed to investigate the upstream/downstream signaling of TH functionally under viral infection, alongside spatiotemporal analyses of Dat acetylation modifications and the key dopamine receptor subtypes mediating altered climbing behavior.

Entomopathogenic fungi initiate infection by breaching the insect’s cuticle; they achieve this through the secretion of chitinolytic, proteolytic, and lipolytic enzymes that break down key structural components like chitin, proteins, and lipids, combined with mechanical pressure [47,48,49]. Once inside, the fungus proliferates within the hemolymph [50]. There, the pathogen compromises host survival by depleting nutrients, secreting toxins, and disrupting the balance of the intestinal microbiota [51]. In contrast, the LdMNPV virus infects *L. dispar* larvae specifically via oral ingestion [52]. Its OBs dissolve in the alkaline conditions (pH 10–11) of the midgut through protease activity, releasing occlusion-derived virions (ODVs) [52,53]. These ODVs then cross the peritrophic matrix (PM) to infect midgut epithelial cells [54]. Infected cells subsequently produce budded virions (BVs) that spread throughout the insect’s body systemically, primarily via the tracheal system [55,56,57]. Although entomopathogenic fungi offer advantages including broad host ranges, environmental safety, and unique killing mechanisms, their application for *L. dispar* control remains underexplored [58,59]. While LdMNPV serves as a key biological control agent against this pest, its utility is constrained by host specificity and slow action kinetics [60,61]. Our findings demonstrate that co-infection yields higher mortality than viral mono-infection while accelerating time-to-death, suggesting a novel integrated pest management approach: strategically combining fungal and viral agents to exploit their complementary mechanisms while mutually compensating for their respective limitations. Despite over 50 commercially registered insect-virus pesticides globally and extensive research on individual entomopathogens, studies on their synergistic deployment remain scarce [62].

Contemporary research on microbial pest control predominantly focuses on efficacy-enhancement strategies. Using baculoviruses as an exemplar, reverse genetics techniques enable the modification of viral genomes to construct expression vectors targeting specific pests [63]. Alternatively, embedding efficacy-enhancing factors into OBs significantly elevates insecticidal efficiency [64]. The establishment of mixed viral populations through co-infection with distinct isolates facilitates the development of “customizable insecticides” capable of simultaneously controlling multiple pest species [65]. Notably, recombinant viruses can infect non-susceptible insects, thereby overcoming host restrictions [66]. Synergistic approaches combining bacteria and viruses have demonstrated enhanced control efficacy. For instance, joint application of Bt and viruses exploits bacterial disruption of host midgut integrity to promote viral entry into the hemocoel, amplifying lethal effects [67]. Experimental results demonstrated that the combined application of the virus and fungus enhanced larval mortality induced by the virus by approximately 50%. Notably, the virulence of the fungus was unaffected by the virus, and fungal activity remained uninhibited even when the virus was applied 24 h prior [68,69]. These findings suggest complementary modes of action between the two pathogens. However, studies on the combined application of entomopathogenic fungi and viruses for the control of *L*. *dispar* remain limited. The co-infection with both pathogens presents a novel approach to the biological control of this pest.

## 5. Conclusions

Preliminary studies on *M. anisopliae* and LdMNPV infecting *L. dispar* larvae under different infection modes revealed a significant difference in the ascent rate between larvae infected solely with the virus and those subjected to fungal–viral co-infection. *M. anisopliae* was found to promote the manifestation of tree-top disease induced by LdMNPV during the early infection stage, while suppressing it during the later infection stage. Concurrently, co-infection experiments demonstrated an accelerated time to death compared to single infections, alongside marked differences in post-infection symptomatology. These findings enhance our understanding of the mechanistic basis underlying tree-top disease in gypsy moths and suggest a potential pest management strategy utilizing combined fungal–viral biocontrol agents.

## Figures and Tables

**Table 1:** Virulence of strain HJN-G3-2C of *M. anisopliae* to larvae of *L. dispar* (10 d).

LC_50_ Value (Spore∙mL^−1^)	95% Confidence (Spore∙mL^−1^)		LC_90_ Value (Spore∙mL^−1^)	95% Confidence (Spore∙mL^−1^)		Regression Equation	χ^2^ _0.05_
	Lower Limit	Upper Limit		Lower Limit	Upper Limit		
1.77 × 10^8^	1.99 × 10^8^	5.06 × 10^10^	2.501 × 10^10^	2.18 × 10^10^	3.12 × 10^13^	Y = 0.406*x* − 2.942	0.485

**Table 2:** Lethal time of strain HJN-G3-2C of *M. anisopliae* and LdMNPV to larvae of *L. dispar*.

Strain	Concentration	LT_50_ Value (d)	95% Confidence (d)		LT_90_ Value (d)	95% Confidence (d)	
			Lower Limit	Upper Limit		Lower Limit	Upper Limit
HJN-G3-2C	5 × 10^9^ spore/mL	3.484	2.907	4.035	11.008	8.916	15.073
	5 × 10^8^ spore/mL	6.905	6.036	8.144	16.218	13.721	24.986
	5 × 10^7^ spore/mL	8.373	7.515	9.746	16.705	13.266	25.392
LdMNPV	2 × 10^8^ OBs/mL	4.910	4.394	5.433	10.619	9.124	13.216
	1 × 10^8^ OBs/mL	5.439	4.951	5.938	10.358	9.092	12.483
	2 × 10^7^ OBs/mL	5.457	4.632	6.385	11.922	9.487	17.921

## Data Availability

The sequences of the *Metarhizium anisopliae* strain HJN-G-3-2C are available in the Genbank database.
